# Differences in Compositions of Gut Bacterial Populations and Bacteriophages in 5–11 Year-Olds Born Preterm Compared to Full Term

**DOI:** 10.3389/fcimb.2020.00276

**Published:** 2020-06-16

**Authors:** Thilini N. Jayasinghe, Tommi Vatanen, Valentina Chiavaroli, Sachin Jayan, Elizabeth J. McKenzie, Evelien Adriaenssens, José G. B. Derraik, Cameron Ekblad, William Schierding, Malcolm R. Battin, Eric B. Thorstensen, David Cameron-Smith, Elizabeth Forbes-Blom, Paul L. Hofman, Nicole C. Roy, Gerald W. Tannock, Mark H. Vickers, Wayne S. Cutfield, Justin M. O'Sullivan

**Affiliations:** ^1^Liggins Institute, University of Auckland, Auckland, New Zealand; ^2^The Broad Institute of MIT and Harvard, Cambridge, MA, United States; ^3^Quadram Institute Bioscience, Norwich, United Kingdom; ^4^A Better Start—National Science Challenge, University of Auckland, Auckland, New Zealand; ^5^Newborn Services, Auckland City Hospital, Auckland, New Zealand; ^6^Malaghan Institute of Medical Research, Wellington, New Zealand; ^7^AgResearch, Palmerston North, New Zealand; ^8^The Riddet Institute, Massey University, Palmerston North, New Zealand; ^9^The High-Value Nutrition Challenge, Auckland, New Zealand; ^10^Department of Microbiology and Immunology, University of Otago, Dunedin, New Zealand

**Keywords:** preterm birth, bacteriophages, metabolomics analysis, gut microbiome, arginine, calprotectin

## Abstract

Preterm infants are exposed to major perinatal, post-natal, and early infancy events that could impact on the gut microbiome. These events include infection, steroid and antibiotic exposure, parenteral nutrition, necrotizing enterocolitis, and stress. Studies have shown that there are differences in the gut microbiome during the early months of life in preterm infants. We hypothesized that differences in the gut microbial composition and metabolites in children born very preterm persist into mid-childhood. Participants were healthy prepubertal children aged 5–11 years who were born very preterm (≤32 weeks of gestation; *n* = 51) or at term (37–41 weeks; *n* = 50). We recorded the gestational age, birth weight, mode of feeding, mode of birth, age, sex, and the current height and weight of our cohort. We performed a multi'omics [i.e., 16S rRNA amplicon and shotgun metagenomic sequencing, SPME-GCMS (solid-phase microextraction followed by gas chromatography-mass spectrometry)] analysis to investigate the structure and function of the fecal microbiome (as a proxy of the gut microbiota) in our cross-sectional cohort. Children born very preterm were younger (7.8 vs. 8.3 years; *p* = 0.034), shorter [height-standard deviation score (SDS) 0.31 vs. 0.92; *p* = 0.0006) and leaner [BMI (body mass index) SDS −0.20 vs. 0.29; *p* < 0.0001] than the term group. Children born very preterm had higher fecal calprotectin levels, decreased fecal phage richness, lower plasma arginine, lower fecal branched-chain amino acids and higher fecal volatile (i.e., 3-methyl-butanoic acid, butyrolactone, butanoic acid and pentanoic acid) profiles. The bacterial microbiomes did not differ between preterm and term groups. We speculate that the observed very preterm-specific changes were established in early infancy and may impact on the capacity of the very preterm children to respond to environmental changes.

## Background

During birth, infants are exposed to their first significant populations of microbes from the mother's perineum, skin and hospital environment. The early stages of microbiome development within the neonatal gut are affected by lengthy hospitalization (Groer et al., 2014; Gregory et al., 2016), inflammatory factors in *utero* (maternal illness, infections, stress) (Walker et al., 2017; Lu and Claud, 2018), antibiotic and steroid exposure to both the mother and infant (Zhu et al., 2017), mode and timing of delivery (Groer et al., 2014), invasive procedures (Groer et al., 2014), type or mode of feeding (i.e., parenteral vs. breast, human milk fortifiers, cow milk based formula) (Hay, 2013; Grier et al., 2017; Zanella et al., 2019), and host genetics (Goodrich et al., 2014, 2016; Bonder et al., 2016; Turpin et al., 2016; Jayasinghe et al., 2017).

The composition of the human gut microbiome (in terms of absolute numbers and the diversity of microbes) develops in complexity after birth, becoming more like that of adults by ~1 year (Bäckhed et al., 2015) before further maturation to an adult-like composition in subsequent years. The transition to an adult-like gut microbiome includes a shift from facultative anaerobic microbes (e.g., *Escherichia coli*, streptococci, enterobacteria and staphylococci and enterococci) to strictly anaerobic microbes (e.g., Ruminococcus *Bifidobacterium, Bacteroides*, and *Clostridium* species) (Turroni et al., 2012; Bergström et al., 2014; Obermajer et al., 2017). However, this represents only a partial window into the complexity of the dynamic populations in the human large intestine. Indeed, recent culture-independent investigations have shown that human microbiomes harbor many unidentified species and most species that can be recovered by metagenomic assembly represent previously unknown taxa (Pasolli et al., 2019). Gut phages are yet another facet of the microbiomes that most studies neglect. Given the preponderance of bacteriophage populations, it is likely that bacteriophage adaptation and transition through infancy represents a further level of regulation (Minot et al., 2013; Lim et al., 2015). However, there is surprisingly little data in humans, and only limited experimental data examining fecal bacteriophage dynamics and impact (Reyes et al., 2010; Minot et al., 2013; Lim et al., 2016; Guerin et al., 2018). In adults, bacteriophage populations are stable over time, but very individual-specific, complicating comparative analyses between individuals and groups (Gregory et al., 2019; Shkoporov et al., 2019).

Being born before 37 weeks of gestation (i.e., preterm birth) is a major life event that correlates with altered patterns of gut colonization (Cilieborg et al., 2012; Groer et al., 2014). Children born very preterm (≤32 weeks gestation) acquire microbes mostly from the environment of the neonatal intensive care unit (NICU), leading to pathogenic colonization of the infant's skin and mucosal surfaces, including the gut (Brooks et al., 2014). Little is known about bacteriophages in the gut microbiota of children born very preterm.

In healthy children, diet changes the gut microbiota in a fairly rapid and easily reversible way (De Filippo et al., 2010) such that there is widely considered to be a general convergence toward a similar population structure between individuals (Rosa et al., 2014). Irrespective of initial differences between the microbiota of infants fed with breast milk or infant formula (Koenig et al., 2011; Timmerman et al., 2017), the introduction of solid weaning foods has a major role in the transition of the early infant into an adult-like microbiome (De Filippo et al., 2010; Bergström et al., 2014).

Changes in the gut microbiome in early life can be associated with the physiology of an individual as the founder microbiome is a determinant of future disease development (Arrieta et al., 2014; Tamburini et al., 2016). These long-term effects could be due to modifications in the gut epithelium, hepatic cells, or immune system that are affected by microbial and host interactions or the presence of microbial metabolites (e.g., folates, indoles, trimethylamine-N-oxide (TMAO), acetic and propionic acids) (Spiljar et al., 2017; Cani, 2018).

The microbes that provide early colonization of the human gut during development lead to a state of “immune education” through toll-like receptors and nucleotide-binding oligomerization proteins (Kawai and Akira, 2009) that contribute to host protection against potential pathogens (Rakoff-Nahoum et al., 2004; Cuenca et al., 2013). The gut microbiota also contributes to patterns of epithelial gene expression, and metabolic programming (Hooper, 2004; Sudo et al., 2004; Neu, 2008).

Here, we performed a cross-sectional multi-omic study on a mid-childhood cohort of children born very preterm and term. The purpose of our study was to determine whether microbial and bacteriophage composition differ between children born very preterm and their term counterparts in mid-childhood.

## Results

### Baseline Characteristics of the Study Cohort

**Figure 1:**
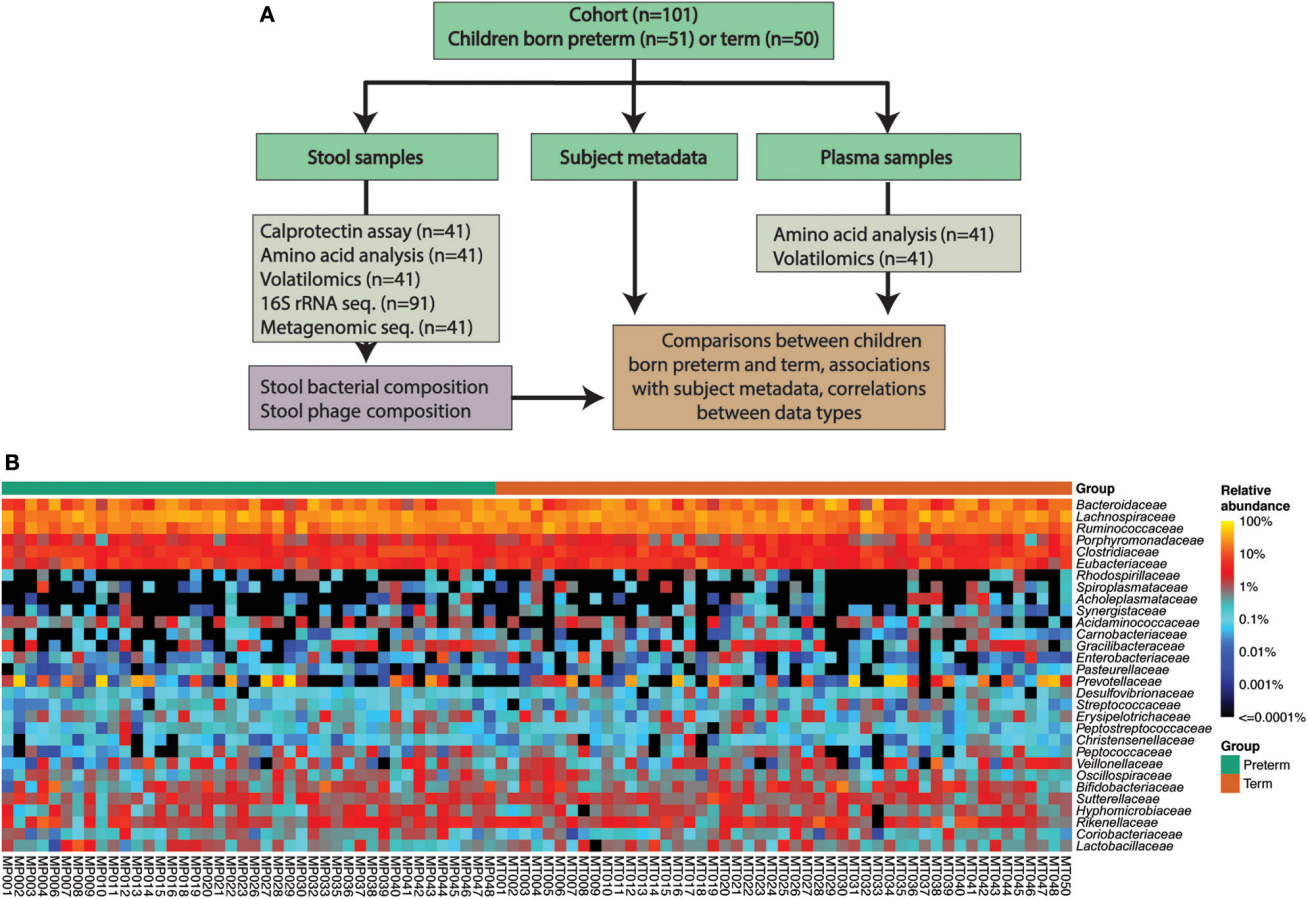
**(A)** Cohort and data overview. **(B)** Abundances of the 30 most abundant bacterial families according to the 16S rRNA amplicon sequencing data.

The cohort consisted of 101 children: 51 born very preterm and 50 born at term (Figure 1A, Table 1). There were auxological differences between the very preterm and term children in our cohort. Children born very preterm were younger, shorter, slimmer, and more likely to be born by Cesarean section than children born at term (Table 1).

**Table 1:** Demography and summary characteristics of the study population.

	**Very preterm children**	**term Children**	***p*-value**
***N***	51	50	
**DEMOGRAPHY**			
Age (years)^a^	7.8 ± 1.3	8.3 ± 1.4	0.034
Sex ratio (females)^b^	20 (39%)	19 (38%)	0.90
Ethnicity (New Zealand European)^b^	39 (76%)	30 (60%)	0.074
**BIRTH CHARACTERISTICS**			
Birth weight standard deviation score (SDS)^a^	0.42 ± 0.91	0.32 ± 0.90	0.60
Gestational age (weeks)^a^	28.1 ± 2.2	39.9 ± 1.2	<0.0001
Delivery (C-section)^b^	30 (59%)	18 (36%)	0.021
**INFANT CHARACTERISTICS**			
Breastfeeding^c^	47 (92%)	48 (96%)	0.68
**CHILDHOOD ANTHROPOMETRY^d^^†^**			
Weight SDS	−0.16 (−0.49–0.10)	0.47 (0.21–0.73)	0.0005
Height SDS	0.31 (0.03–0.58)	0.92 (0.67–1.17)	0.0006
BMI SDS	−0.20 (−0.40–−0.01)	0.29 (0.09–0.49)	<0.0001

*Age data are means ± standard deviation; categorical data are n (%)*.

a One-way ANOVA;

b Chi-square test;

c Fisher's exact test;

d *SDS outcomes: general linear regression models adjusted for sex, ethnicity and birth order, as well as mean parental BMI or mid-parental height*.

† *Data on anthropometry is estimated marginal means and respective 95% confidence intervals, adjusted for confounding factors*.

### Significant Differences in Bacterial Microbiota Between 5-11-Year-Old Children Born Very Preterm and at Term Were Not Observed

16S rRNA gene amplicon sequencing was performed for samples from 91 individuals (*n* = 42 born preterm; *n* = 49 born term) that provided stools (Figure 1B). We identified 123 bacterial families, 139 genera and 322 species across 91 children (42 very preterm and 49 term). Bacterial composition at the species, genus and family levels, were not different between the two study groups according to an analysis of the Linear discriminant (LDA) effect size (Segata et al., 2011) (Figure 1B). Measures of alpha diversity, an estimate of observed species (chao1) and Shannon and Simpson indices were not different between groups (Supplementary Figure 1). Principal coordinates analysis (PCoA) of beta diversity (i.e., Bray-Curtis dissimilarity) confirmed that the gut bacterial communities of children born very preterm and at term were not different at species, genus or family level (PERMANOVA, *p* > 0.05, Supplementary Figure 2). PCoA analyses also confirmed that other potential confounders (i.e., sex, ethnicity, mode of delivery, intake of antibiotics since hospital discharge, and mode of feeding [breast vs. formula]) did not demonstrably impact microbial diversity at species, genus or family level, as measured by 16S rRNA gene amplicon sequencing (PERMANOVA, *p* > 0.05, Supplementary Figures 2B–F).

**Figure 2:**
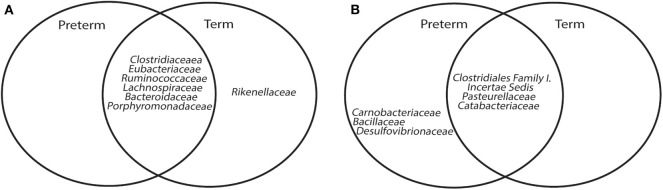
Venn Diagrams of major **(A)** and minor **(B)** core microbiome of children born very preterm and at term.

A core microbiome analysis (CORBATA) (Li et al., 2013) was performed on the 16S rRNA gene amplicon data to determine the ubiquity and evenness of the distribution of bacteria across our cohort (*n* = 91). Children born very preterm and at term share *Clostridiaceae, Eubacteriaceae, Ruminococcaceae, Lachnospiraceae, Bacteroidaceae*, and *Porphyromonadaceae* families as the major components [i.e., ubiquity score of ≥ 80% and relative abundance of ≥1% (Li et al., 2013)] within their core microbiomes (Supplementary Figure 3). Notably, the core microbiome of children born at term also contained members of the *Rikenellaceae* family (82 and 1% for ubiquity and relative abundance, respectively; Supplementary Figure 3). In contrast to our observation of the major core, the minor core (i.e., ubiquity score ≥50% and relative abundance of <1%) of the term group (*Pasteurellaceae, Catabacteriaceae*, and *Clostridiales Family I. Incertae Sedi*) was a subset of the very preterm minor core, which also contained *Desulfovibrionaceae, Bacillaceae*, and *Carnobacteriaceae* (Figure 2A, Supplementary Figure 4). Despite the observed differences between the major and minor cores within the microbiomes of children born very preterm and term, the Abundance-Weighted Kolmogorov-Smirnov statistic indicated that they were not different at a *p*-value of 0.05 from each other at family, genus or species level (Figure 2B, Supplementary Figure 5). Collectively, these results demonstrated that the bacterial components of the gut microbiota in the very preterm and term born children were not significantly different at the level of the 16S rRNA amplicon analysis. This does not preclude the possibility of strain level differences between these cohorts.

### Children Born Very Preterm Have Low-Grade Gut Inflammation

We randomly selected a subgroup of 41 children [*n* = 20 very preterm [7 females and 13 males with a birth weight <1,500 g] and *n* = 21 term [11 females and 10 males]] for multi-omics analysis (i.e., metagenomic sequencing and discovery volatilomics [SPME GC-MS]; methods) to characterize any metabolic differences or functional shifts in the gut microbiomes. Baseline characteristics of the subgroup are provided in Supplementary Table 1.

**Figure 3:**
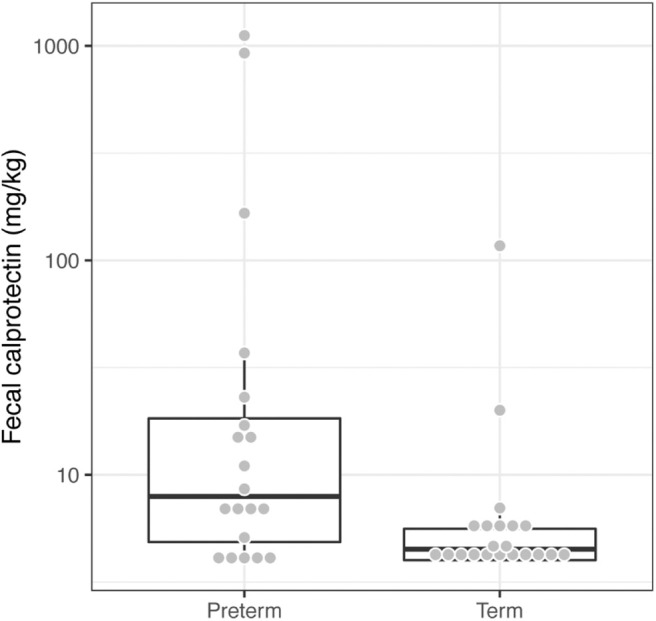
Children born very preterm had higher fecal calprotectin levels compared to term children (Wilcoxon test, *p* = 0.0064). The fecal calprotectin concentrations, y-axis, are shown on a logarithmic scale.

We measured fecal calprotectin levels as a proxy for gut inflammation. Children born very preterm had higher fecal calprotectin levels in comparison to their term counterparts (Wilcoxon test, *p* = 0.0064, Figure 3). This suggests that the low-grade inflammation previously described in preterm babies (Weaver et al., 1984; Shulman et al., 1998; van Elburg et al., 2003) potentially persists into mid-childhood.

### There is a Reduction in Gut Phage But Not Bacterial Richness in Children Born Very Preterm

We surveyed the same subset of 41 samples using metagenomic and metabolomic analyses to obtain a more detailed view of the gut microbiome structure and the molecular environment in the gut. To test for differences in the bacterial metagenomes between children born at term or very preterm, we first constructed bacterial profiles using a reference genome-based method (Supplementary Figure 6) (Truong et al., 2015). We did not observe a difference in the overall microbial richness between the groups, measured by the total number of detected bacterial species (Wilcoxon rank sum test *p* = 0.78). Furthermore, microbial taxonomic composition or functional potential determined using whole metagenome sequencing did not differ between the two study groups according to LDA. Complementing the reference-based approach, we assembled metagenomic contigs and detected a total of 2,112,701 non-redundant gene families. We identified no differences between very preterm and term children in either gene richness (Wilcoxon rank sum test *p* = 0.36), or in the number of assembled contigs per individual. We acknowledge that our study is not well-powered to detect changes between the groups, with a probability of 67% to detect differences of one standard deviation between the groups (Student's *t*-test power calculation, *n* = 20 subjects per group, effect size = 1 standard deviation, alpha = 0.01).

**Figure 4:**
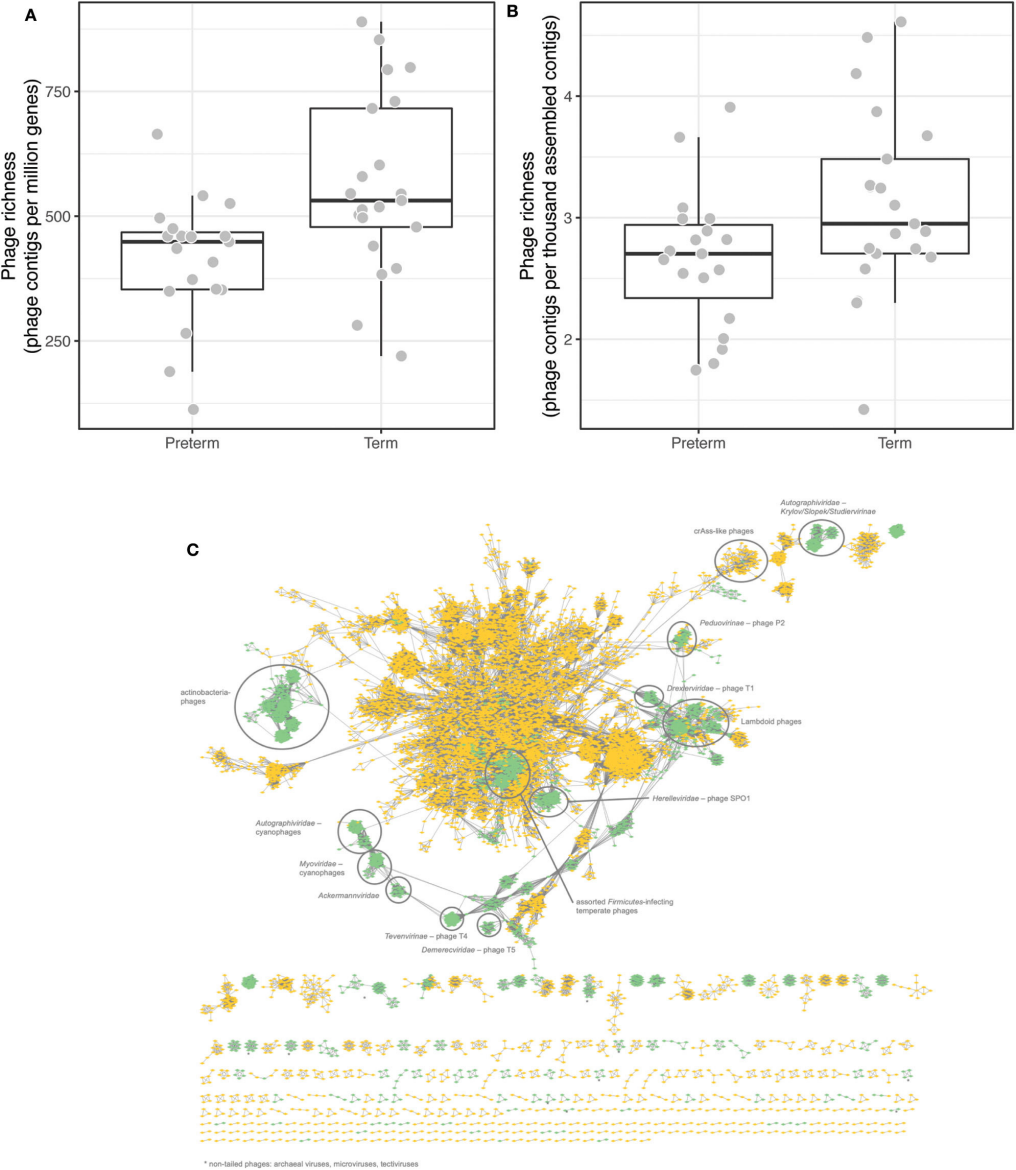
Gut phage richness differed between children born preterm or term. **(A)** Phage richness measured by the number assembled partial phage genomes per million genes; mean values 412 and 536 partial phage genomes per million genes in children born preterm and term, respectively. **(B)** Phage richness measured per thousand assembled contigs; mean values 2.66 and 3.11 partial phage genomes per thousand assembled contigs. **(C)** Gene-sharing network of assembled phage genomes (from all 41 individuals in the subgroup of this study) and reference genomes from Viral RefSeq v94 annotated with selected members of a subset of clusters. Reference genomes are in green, partial phage genomes from this study in yellow.

Bacteriophages (phages), viruses specific to bacteria, are able to reshape bacterial communities and may also interact with mammalian immune systems (Van Belleghem et al., 2018). To complement our analysis of the bacterial microbiomes in this under-powered setting, we estimated the number of partial phage contigs in the assembled metagenomes to test for any differences in phage richness. We observed higher phage richness, i.e., more assembled partial phage contigs, in term compared to the very preterm children, while adjusting for: (1) the number of observed genes (Figure 4A, Student's *t*-test *p* = 0.0039, Wilcoxon rank sum test *p* = 0.0022); or (2) the total number of assembled contigs (Figure 4B, Student's *t*-test *p* = 0.038, Wilcoxon rank sum test *p* = 0.036).

We co-clustered the 10,182 partial phage genomes, from all 41 individuals in the subset, by gene-sharing network analysis in vContact2 (Bin Jang et al., 2019). Phage genomes detected in this study fell into 1,161 distinct viral clusters (VCs) roughly representing genus-level taxonomic groups. VC richness differed between the groups mirroring what was seen for the partial phage genomes (Supplementary Figure 7). Strikingly, only 13 of these VCs obtained family level, and 4 VCs obtained genus level annotations through existing RefSeq annotations, leaving the majority of the phage genomes observed here unannotated illustrating the high prevalence of unknown phages in the gut communities (Figure 4C). 0.4% (43/10,182) and 0.1% (12/10,182) of the phage genomes obtained annotations at the family and genus levels, respectively. No VCs showed significantly different prevalence (test of proportions) between children born very preterm and term.

### Fecal and Plasma Metabolite Profiles Differ in Children Born Very Preterm and at Term

**Figure 5:**
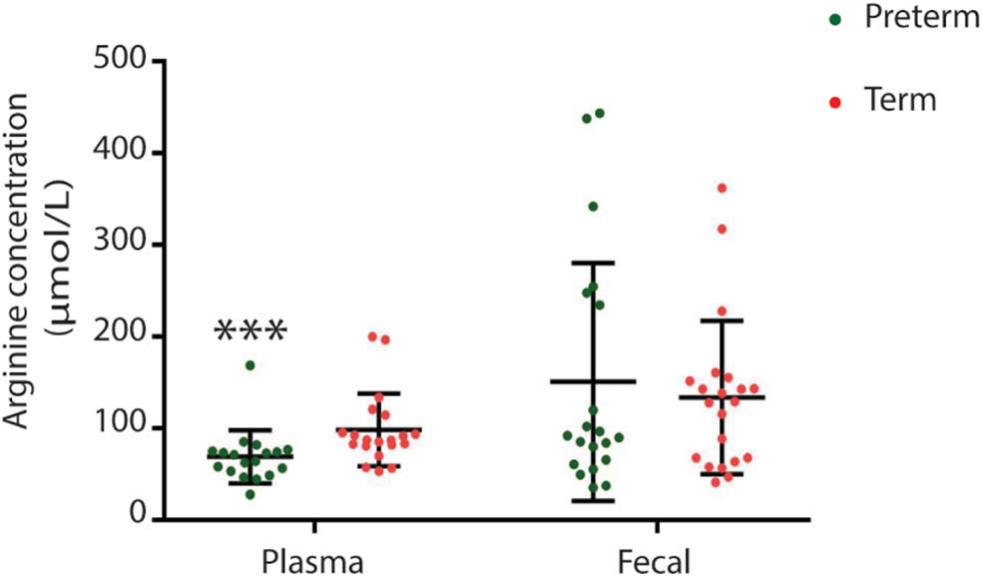
Plasma but not fecal arginine levels were lower in children born very preterm (***p = 0.0007).

**Figure 6:**
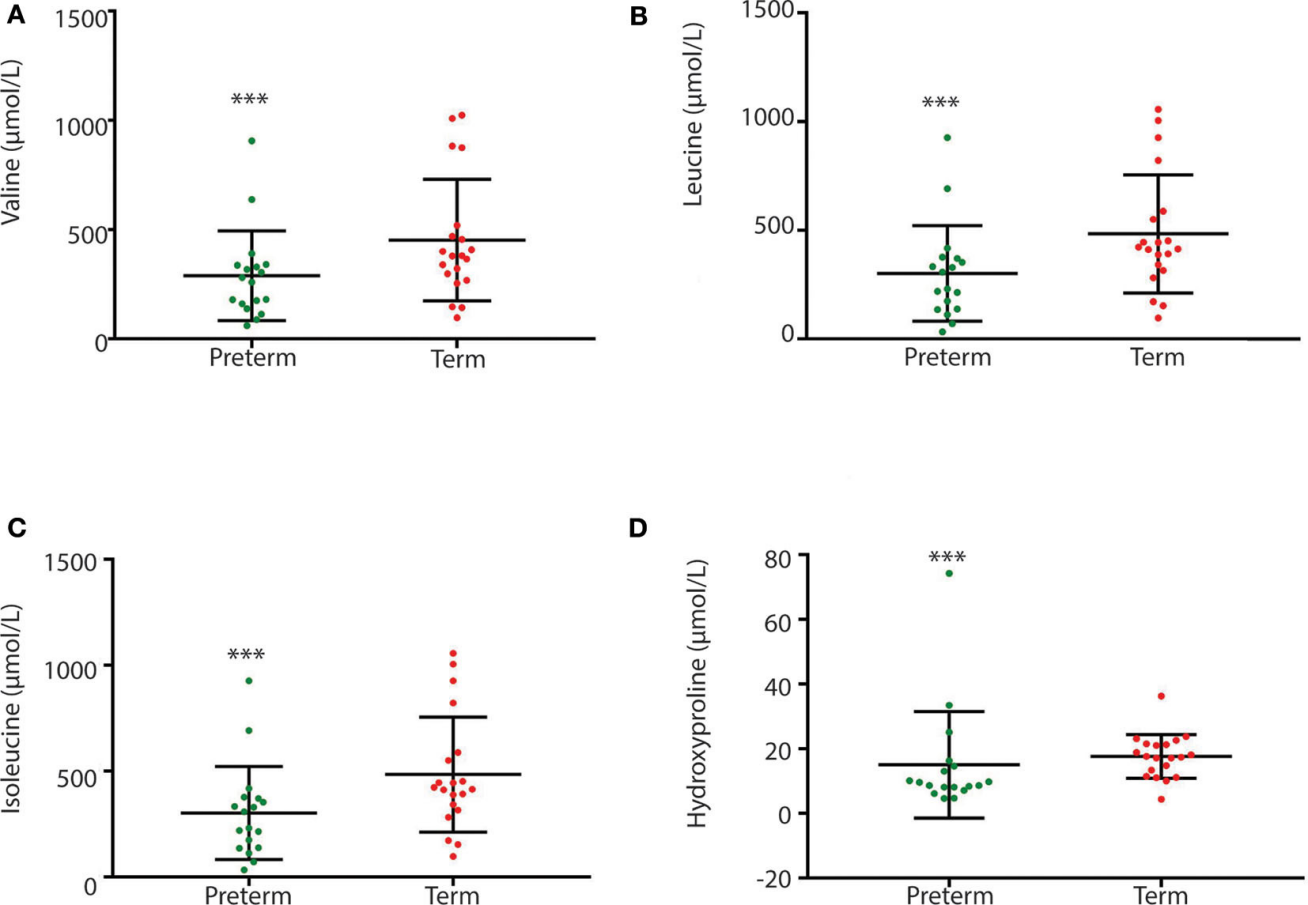
Between group variation of fecal branch chain amino acid and hydroxyproline concentrations (μmol/L) **(A)** valine; **(B)** leucine; **(C)** isoleucine; and **(D)** hydroxyproline. Concentrations were significantly different between children born very preterm (*n* = 18) and children born at term (*n* = 20). [***p-values are 0, 0.022, 0.008, 0.003, and 0.007, respectively (Unpaired *t*-test; non-parametric Mann-Whitney test, GraphPad Prism 7.03)].

Children born very preterm had lower plasma but not fecal arginine concentrations when compared to children born at term (*p* < 0.001; Figure 5). Fecal arginine levels were more variable in the very preterm children. However, the majority of arginine values were <100 μmol/L in the very preterm children whereas the majority of values were >100 μmol/L in term children. Amino acid quantitation identified lower fecal levels of branched-chain amino acids in children born very preterm than children born at term together with hydroxyproline (*p* = 0.022, *p* = 0.008, *p* = 0.003, and p = 0.007 for valine, leucine, isoleucine and hydroxyproline, respectively; Figure 6).

**Figure 7:**
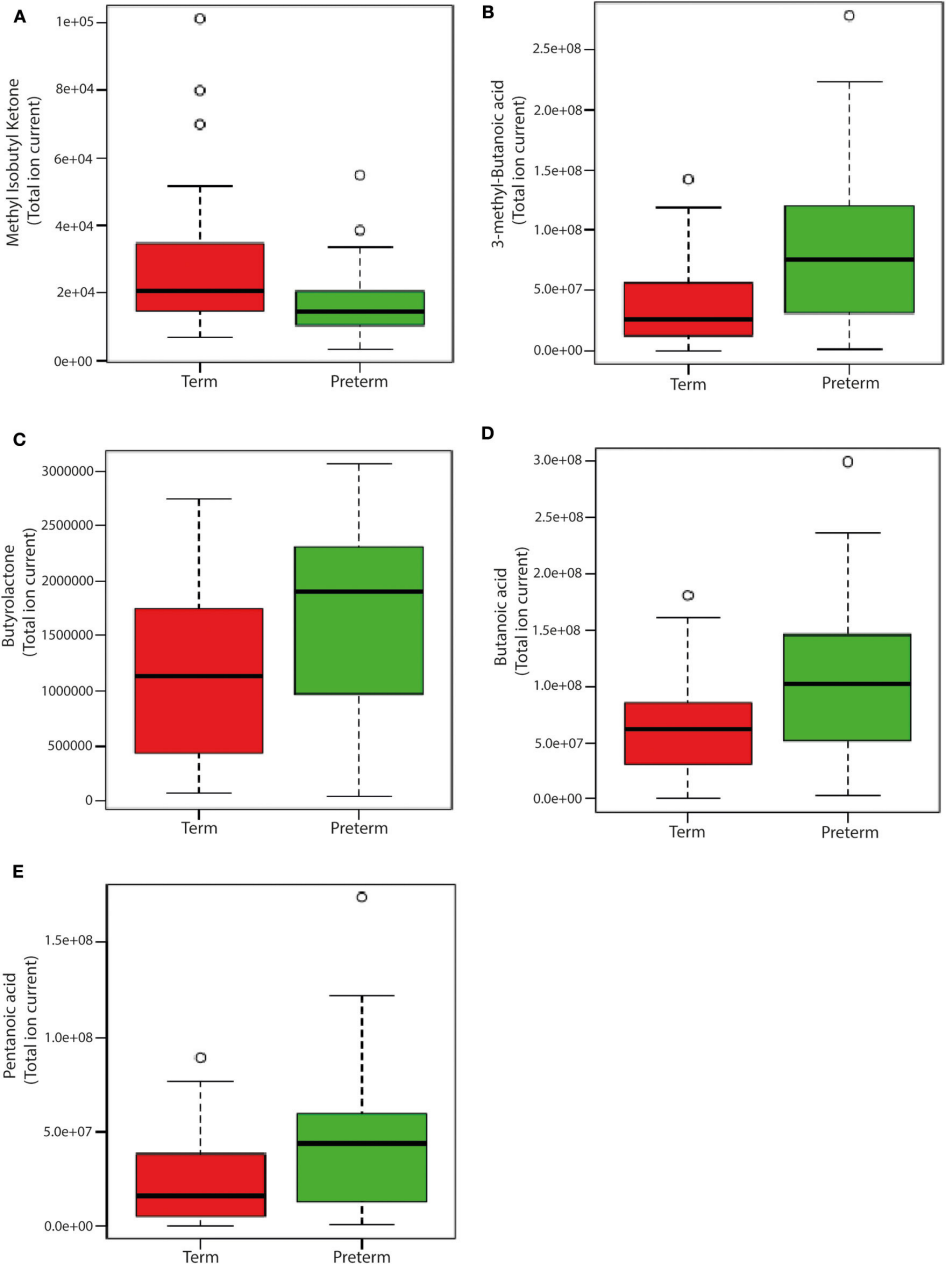
Box-whisker plots of significantly different fecal volatiles in children born very preterm and term. **(A)** Methyl Isobutyl Ketone (*p* = 0.0297); **(B)** 3-methyl-Butanoic acid (*p* = 0.0297); **(C)** Butyrolactone (*p* = 0.0356) **(D)** Butanoic acid (*p* = 0.0356); and **(E)** Pentanoic acid (*p* = 0.0356). The analysis was performed on MetaboAnalyst 3.0 (a web server for metabolomics data analysis and interpretation) (Xia et al., 2009). Two groups [very preterm (*n* = 20) and term (*n* = 21)] were compared using unpaired *t*-test (Wilcoxon Mann Whitney test) with FDR correction using Bonferroni correction and Holm step-down procedure on MetaboAnalyst 3.0. Fecal volatile data is presented as relative abundance and the measure is the peak area of the most intense ion for that molecule. Unit is given as “total ion current”.

Levels of 3-methyl-butanoic acid, butyrolactone, butanoic acid and pentanoic acid were higher (*p* < 0.05, Figure 7) in the feces of children born very preterm than those born at term. By contrast, fecal levels of methyl isobutyl ketone were higher in children born at term. In addition to these specific metabolites, there were an additional 34 plasma volatiles that were different between groups (*p* < 0.05; Supplementary Table 2).

## Discussion

We identified a decrease in the phage strain-level richness present in feces in infants born very preterm. While studies describing the bacteriophage component of the gut microbiome are scarce; bacteriophage and bacterial richness are inversely correlated in an age-dependent manner from birth (Lim et al., 2015) before becoming relatively stable in the adult intestine (Reyes et al., 2010; Minot et al., 2013). It has been postulated that the high bacteriophage concentrations observed at birth collapse because of low bacterial colonization density, which allows the gut to be colonized and drives a shift in bacteriophage composition (Lim et al., 2015). Differences in the microbial population observed in preterm infants over first months of life would result in a greater and more sustained bottleneck in the bacteriophage population. Such a scenario may explain our observations, but only if intra-phage competition occurs to reduce co-infection rates and prevent bacteriophage population expansion. Despite this, the impact of phage competition is not obvious given our inability to identify the majority of the phage sequences we isolated. As such, definitive evidence linking the bacteriophage population structure to the preterm phenotype and ability to adapt to environmental change (e.g., diet, stress, or illness) remains uncertain.

We did not identify significant differences in the bacterial communites of the very preterm children in mid-childhood when compared to term children. This observation was confirmed by both 16S amplicon or shot-gun metagenomic sequencing. Superficially, this finding is counter intuitive given the large scale alterations that have been identified from early cross-sectional analyses, at periods ranging from <2 weeks until ~20 months of age, of the gut microbiome of children born very preterm (<32 weeks of gestational age) (Aagaard et al., 2014; Groer et al., 2014; DiBartolomeo and Claud, 2016; Patel et al., 2016; Chernikova et al., 2018). However, our results are consistent with published observations of convergence of the gut microbiome population following neonatal development (de Muinck and Trosvik, 2018). Therefore, it appears that the developmental trajectory of the bacterial component of the microbiome into childhood is resilient to early-life stress. This supports the hypothesis that changes during neonatal development (e.g., development of anoxia in the gut, breast feeding and weaning [*e.g.*, (Leong et al., 2018)]) make a major contribution to the microbial population phase transitions that occur. This argues that acceleration of these transitions (e.g., introduction of breast milk to preterm babies at an appropriate gestational age) will have short term impacts on microbial population development. Randomized controlled longitudinal prospective studies of this hypothesis should be performed.

Arginine is a conditionally essential amino acid that is obtained from food or synthesized from glutamate via citrulline (Tomlinson et al., 2011). Decreased plasma arginine levels in children born very preterm in this cohort may reflect long-term reductions in host arginine biosynthetic capacity or altered bacterial synthesis. This result is in direct contrast to that observed by Posod et al. who observed an increase in the plasma arginine levels in 5-7 years old children born <32 weeks of gestation (Posod et al., 2017). It is possible that the observed disparity is due to differences in the clinical treatment (duration and composition of parenteral nutrition) (Yarandi et al., 2011) as no clinical trials have looked at the long-term impact of preterm parenteral feeding on plasma amino acid levels in infants. Notably, children born very preterm have a reduced capacity to synthesize arginine due to limited expression of arginine biosynthetic genes (e.g., pyrroline-5-carboxylate synthase, argininosuccinate synthase, and lyase) (Wu et al., 2004).

We contend that the observed relative increase in calprotectin levels in children born very preterm is associated with subtle gut inflammation and altered permeability. The higher plasma metabolite levels in children born preterm are consistent with impaired gut epithelial integrity reducing the selectivity of transfer of gut microbiome metabolites into the bloodstream. For example, elevated levels of microbial derived succinate are correlated with plasma levels of zonulin, a marker of intestinal permeability and low grade inflammation, in human obesity (Serena et al., 2018). Children born very preterm have been reported to exhibit altered gut permeability during early life (Weaver et al., 1984; Shulman et al., 1998; van Elburg et al., 2003), possibly in response to interrupted gut epithelial development *in utero* or increased enteral feeding (Rouwet et al., 2002). Importantly, ongoing low-grade inflammation is a known risk factor for development of some chronic diseases that preterm children have an increased susceptibility to such as diabetes (Hofman et al., 2004; Kajantie et al., 2010; Crump et al., 2011a), hypertension (Crump et al., 2011b), cardiovascular disease (Carr et al., 2017), and asthma (Crump et al., 2011c).

Recent longitudinal multi-omics studies in prediabetic adults (25–75 years) identified diverse patterns of intra- and inter-personal variability at all levels of the healthy profile (including the microbiome) and changes in fecal calprotectin that are not associated with the fecal microbial compostion (Zhou et al., 2019). Consistent with this, we observed higher fecal calprotectin levels in the very preterm group that were accompanied by lesser gut bacteriophage richness and changes in plasma and fecal metabolites but not in gut bacterial populations.

While the multiple ‘omics techniques we used provided insights into differences between children born preterm and term, this study also has some important limitations. Since all biological samples were collected 5–11 years after birth, we do not know whether the differences between the study groups arose in early life or later. These differences could also be caused by unobserved external factors that may, or may not be, linked to lifestyle differences between the study groups. The bioinformatics of the metagenomic and 16S sequencing data we used rely largely on reference databases. Since viral and phage reference collections only capture a small fraction of gut viral diversity, we could not assign any taxonomy for the majority of the assembled partial phage genomes. Similarly, bacterial microbiomes contain a smaller but significant portion of unknown organisms. There may be further trends among yet unknown taxonomic groups that were therefore left unobserved. Metabolomic and volatilomic analyses are also highly dependent on known compounds and their spectra, and typically result in the identification of only a miniscule portion of the highly diverse stool metabolome. Finally, this study is agnostic of the absolute bacterial or phage biomass. Quantifying microbial cells and viral particles by, for example, quantitative PCR, flow cytometry or microscopy, could provide additional insights in phage-bacteria relationships in future studies.

While potential early differences in bacterial microbiota were lost over time, we observed reduced phage richness–even after 5–11 years–in children born preterm. We hypothesize that the reduction in phage community richness was due to developmental differences in the microbial population in the neonatal preterm environment. Moreover, we speculate that these changes may impact on the ability of the preterm children's microbiota to respond to acute environmental changes. Future longituginal studies of microbiome and child development should therefore incorporate bacteriophage analyses to more comprehesively profile the human holobiont.

## Methods

### Study Design

We recruited 101 healthy prepubertal children (5–11 years of age) born very preterm (≤32 weeks of gestation) or at term (37–41) weeks of gestation. Children were excluded from the study if: (1) they were conceived by *in vitro* fertilization; (2) their mother had gestational diabetes; hypertension; chronic illnesses; (3) they were born small, or large for gestational age; (4) they were part of a multiple birth; (5) they had a history of bowel surgery; (6) they took antibiotics, probiotics, or oral steroids in the previous month; (7) they had first-degree relatives with type 2 diabetes mellitus; (8) they had siblings in the study; (9) they suffer from chromosomal abnormalities or syndromes, developmental disorders, ongoing significant chronic illness; and (10) they showed signs of puberty (i.e., Tanner stage 2 breast development in girls, and testicular volume >3 mL in boys, or evidence of adrenarche).

A subgroup of 20 children born very preterm and 21 children born at term was randomly selected for shot-gun metagenome, and SPME-GCMS metabolomics profiling. The subgroup of children was chosen randomly, with the very preterm children group being chosen from those with a birth weight <1,500 g.

### Medical History and Demographic Data Collection

The study protocol was approved by the Southern Health and Disability Ethics Committee (Ministry of Health, New Zealand 14/STH/90). Written consent was provided by children's parents. Medical history was obtained from hospital records and parental recall (e.g., duration of breastfeeding). Medical history included pregnancy and delivery history and medications, delivery mode, auxology at birth, gestational age, birth order, and duration of breastfeeding (Table 1). Birth weight data were transformed into standard deviation scores (SDS) (Cole et al., 1995).

Ethnicity data were self-reported using a prioritized system: if multiple ethnicities were selected, children were allocated to a single ethnic group according to a hierarchical classification (Statistics New Zealand, 1997).

### Assessments

All assessments were performed at the Maurice and Agnes Paykel Clinical Research Unit (Liggins Institute, University of Auckland). Each child was assessed during a single visit following an overnight fast.

### Anthropometric Measurements

Children's heights were measured using a Harpenden's stadiometer to the nearest mm, and transformed to SDS based on Tanner/Whitehouse reference data (Tanner and Whitehouse, 1976). Weight was assessed on Seca scales. Body mass index (BMI) was calculated, and transformed to SDS using UK derived reference curves (Cole et al., 1995).

### Stool Sample Collection

Stool samples were collected from 49 children born at term and 42 children born very preterm children. Following passage, samples were transferred to tubes with and without 2 mL of RNAlater (Qiagen) for metagenomic and metabolomics analysis, respectively. Samples when then immediately frozen at−20°C in the participant's home freezer or transported directly to the Liggins Institute (Auckland University) within 2 h where they were frozen (−80°C). Nine children born very preterm and one child born term did not provide stool samples.

### Fecal DNA Extraction

All DNA isolations were performed in a disinfected U.V. sterilized class II hood at room temperature. Microbial genomic DNA was extracted according to Giannoukos et al. (2012) with slight modifications. Briefly, stool samples collected in RNA*later* (Qiagen) were divided into two ~100 mg aliquots. Both aliquots were sedimented (10 min, 14,000 rpm, room temperature) and the supernatant discarded. The pellet was resuspended in bacterial lysis buffer [100 μl; 7.5 mg lysozyme (20,000 units/mg dry weight) 1 μL 0.1 M EDTA, 15 μL 1 M tris-buffer and 484 μL water] and 10 μL proteinase K added (20 mg/mL) (Qiagen). Samples were incubated (10 min, room temperature) with vortexing (30 s every 2 min) and treated with 1.2 mL RLT Plus buffer (Qiagen) and 12 μL beta-mercaptoethanol (Sigma-Aldrich). Acid washed glass beads [1 ml; ≤106 μm (−140 U.S. sieve) (Sigma-Aldrich)] were added to each sample and shaken vigorously (30 Hertz frequency, 10 min) on a TissueLyzer II (Qiagen). The supernatant was removed and added to a QIAshredder spin column (Qiagen) and centrifuged (9,000 rpm, 2 min, room temperature). The eluent was added to an AllPrep DNA (Qiagen) spin column and centrifuged (30 s, 14,000 rpm, room temperature). The AllPrep DNA spin column was used for DNA extraction according to the manufacturer's instructions. Finally, DNA was eluted with EB buffer and aliquots stored at −80°C. Purified water was extracted as a negative control.

DNA concentrations were quantified using a NanoDrop 1000 spectrophotometer (Thermo Fisher Scientific). The average A260/A280 ratio for DNA was 1.79 and A260/230 ratio was 0.98. Concentrations of DNA were subsequently measured using a Qubit® dsDNA HS (double strand DNA high sensitivity) assay. The average yield of DNA was 15.8 μg. The negative control samples did not contain detectable amounts of DNA and were excluded from downstream analysis.

### 16S rRNA Gene Amplicon Sequencing

The Ion 16S™ Metagenomic kit (Thermo Fisher Scientific) was used to produce 16S rRNA gene amplicons from 3 ng of each microbial DNA sample (*n* = 91; 42 very preterm and 49 term children) according to the manufacturer's instructions. Briefly, primer set 1 amplified the V2-4-8 regions whilst primer set 2 amplified the V3-6, 7-9 regions of the bacterial 16S rRNA genes. Eighteen amplification cycles were performed to minimize the PCR bias. Following amplification, 20 μL from each primer-amplicons mix was combined and purified (Agencourt® AMPure® XP beads; Beckman Coulter life sciences). Purified short amplicons were end repaired using the Ion Plus Fragment Library kit (Thermo Fisher Scientific). End repaired amplicons were adaptor ligated using adapters 1 to 16 (Ion Xpress™ barcode adaptors 1-16 kit, Thermo Fisher Scientific), nick repaired, and purified. Purified, adaptor-ligated libraries were amplified (5 PCR cycles) using the Ion Plus Fragment Library kit (Thermo Fisher Scientific). Amplified libraries were purified (Agencourt® AMPure® XP beads) and stored (−20°C) for subsequent analysis.

### Metagenomic Sequencing

Genomic DNA was fragmented using a M220 Focused-ultrasonicator (Covaris, Massachusetts). Fragmentation was performed for 375 s at 20°C temperature producing ~100 bp DNA fragments. Genomic libraries were constructed using Ion Xpress™ Plus Fragment Library Kit and Ion Xpress™ Barcode Adapters 1-16 (Thermo Fisher Scientific, Delaware).

### Sequencing of 16S rRNA Gene Amplicon and Metagenomic Libraries

The molarity (pmol/L) of each purified amplified library was determined on an Agilent 2100 Bioanalyzer® (Agilent technologies) using a high sensitivity DNA kit. Libraries were diluted to a concentration of 26pM using low TE buffer (Ion Plus Fragment Library kit). Diluted libraries were pooled in groups of eight to obtain a 25 μL of final volume for Ion 318™ Chip templating (Thermo Fisher Scientific) on the Ion Chef™ Instrument (Thermo Fisher Scientific).

16S RNA gene amplicon sequencing was performed on a Ion Personal Genome Machine® (PGM™) System using the Ion 318™ Chip v2 and Ion PGM™ Sequencing 400 Kit v2 (Thermo Fisher Scientific). All the data were directed to the Ion PGM server and sequences were sorted according to the barcode. We obtained an average of 897,182 16S rRNA amplicon reads per sample.

### Analysis of 16S rRNA Sequencing Data

16S rRNA gene amplicon data analysis was performed using the Ion Reporter™ software using the default parameters (Supplementary Table 3). The Ion Reporter software performed two-step Basic Local Alignment Search Tool (BLAST) alignment of sequencing reads to the MicroSEQ® (Thermo Fisher Scientific) and Greengene databases (Life Technologies, 2014). Following processing, the obtained amplicon sequences were subsampled at a read depth equal to the sample with lowest sequencing coverage (159,464 reads per sample). Rarefaction curves for observed taxa at species, genus and family level are shown in Supplementary Figure 8. Completed analyses provided results by primers or consensus and visualization graphs for six taxonomic levels (i.e., species, genus, family, order, class, and phylum). The Ion Reporter software also calculated alpha and beta diversity measures for the samples.

### Metagenomic Sequencing Bioinformatics

Metagenomic reads of each sample were assembled into contigs using MegaHIT (Li et al., 2015) (default settings) and contigs shorter than 500 bp were filtered out from downstream analyses. Open reading frames were predicted using Prodigal (metagenomic mode; -p meta) (Hyatt et al., 2010). A non-redundant gene catalog was constructed by clustering genes based on sequence similarity at 95% identity and 90% mutual coverage using CD-HIT (Fu et al., 2012). Metagenomic gene counts were estimated by mapping quality trimmed reads from each sample to the gene catalog with Burrows-Wheeler Aligner (BWA-MEM algorithm, default settings) (Li and Durbin, 2009). The number of partial phage genomes per sample was estimated using VirSorter v1.0.5 (database v2) (Roux et al., 2015) which accurately detects viral genomes using viral hallmark genes and several secondary metrics. The total number of predicted phages (Categories 1–3) was used when comparing phage richness between term and preterm children. To account for sequencing depth, we measured phage richness per the number of observed genes and the number of assembled contigs. Partial phage genomes were further co-clustered with viral genomes from Prokaryotic RefSeq genome collection (version 94) using vContact2 v0.9.10 (with arguments “–rel-mode ‘Diamond’ –pcs-mode MCL –vcs-mode ClusterONE”) (Bin Jang et al., 2019). Any taxonomic identities from phage genomes co-clustering with annotated RefSeq genomes were propagated to partial phage genomes observed in these data.

### Fecal and Plasma Volatilomics

Frozen feces (~200 mg) were weighed out from 4 l fecal samples (20 very preterm + 21 term) into headspace vials in a class II biohazard hood. For the 41 plasma samples (matched to the fecal samples), 300 μL was transferred into headspace vials. Vials were maintained on dry ice throughout the process to limit loss of volatiles, and were stored at−80°C until analysis.

Extraction of short chain fatty acids and other volatile compounds from feces and plasma was carried out using Solid-Phase MicroExtraction (SPME) (Pawliszyn, 1997; Julák et al., 2003; Garner et al., 2007; De Lacy Costello et al., 2008; Grice et al., 2009; Bianchi et al., 2011; Dixon et al., 2011; De Angelis et al., 2014; Mayor, 2014; Stahl et al., 2015; Hough et al., 2018). A StableFlex™ 1 cm, 50/30 μM DVB/Carboxen/PDMS fiber (Sigma-Aldrich) was chosen for its broad volatility and polarity range. Samples were randomized for analysis, blocking for very preterm vs. term. Fecal samples were incubated at 37°C for 20 min and the SPME fiber was exposed to the headspace above the sample for 20 min. The plasma samples were agitated and incubated for 10 min, then exposed to the SPME fiber for 10 min.

Samples were analyzed on a Shimadzu QP2010 Plus Gas Chromatograph Mass Spectrometer using instrument grade helium (99.99%, BOC) as the carrier gas. The SPME fiber was desorbed in the GC injector in splitless mode, in a SPME-specific glass liner (0.75 mm ID) at 250°C for 1 min. A general purpose column (i.e., Rtx-5Sil MS 30 m, 0.25 mm ID) was chosen for compound separation (Shimadzu). Column flow was set at 1 mL/min.

The GC thermal program began isothermally at 40°C for 2 min; increased 10°C/min to 160°C; 5°C/min to 200°C; 20°C/min to 300°C and held 3 min, to give a total run time of 30 min. The detector source was set at 200°C and the quadrupole at 150°C. The detector voltage was 70 eV. Data was acquired at 2000 amu/s in scan mode, with a mass range of 30 - 400 amu.

### SPME GC-MS Data Extraction

Deconvolution and identification of compounds was performed using the Automated Mass Spectral Deconvolution and Identification System (AMDIS Version 2.71) (Stein, 1999). The AMDIS limitation on mass spectral library size was circumvented by developing smaller subset libraries from the 2014 National Institute of Standards & Technology (NIST) main mass spectral library (NIST Standard Reference Database, 1A v17 | NIST) using Agilent MSD Productivity ChemStation (Version F.01.01.2317). The top five identities for each peak, for all peaks, for all samples were combined to form subset libraries of 20,658 mass spectra. The settings for AMDIS were optimized to maximize annotation of all components, slightly increasing the false positive rate, but reducing the false negative rate to <5%.

Mass spectral matching was used to assign identities. Matches <60% were considered unknown. Matches 60–80% were considered identified to the compound class level, and those >80% were considered putative identifications (Sumner et al., 2007). An R-script incorporating XCMS (Smith et al., 2006) was used for peak integration. Data produced represents a set of probable identifications for each feature, filtered so that one identification is assigned to each feature except where co-elution was apparent. Negative controls (empty vials) were used to identify and remove background contaminants.

Two replicates (2 × 200 mg sample) were analyzed from each sample. Sample replicates underwent statistical testing together as an averaged value. Data acquired represents the relative abundance of volatile compounds in 41 subjects.

### Fecal Calprotectin Assay

Calprotectin from fecal samples (20 very preterm + 21 term) was extracted using an EliA™ Stool Extraction kit. The extraction tubes were pre-filled with 750 μL of EliA calprotectin extraction buffer. The rod that was attached to the extraction tube cap was inserted into the stool sample until the four notches were completely covered with stool. The rod was then inserted back into the extraction tube before homogenization of the sample within the extraction buffer for a few seconds. Homogenized samples were incubated (10 min), the homogenate transferred to an Eppendorf tube, and centrifuged (5 min, 3,000 × g). The supernatant was transferred to a fresh tube which was used for calprotectin testing. Fecal calprotectin was determined by the EliA™ Calprotectin assay 2 on Phadia® 250 (Thermo Fisher Scientific) according to the manufacturer's instructions by Abacus ALC (Auckland, New Zealand).

### Plasma Amino Acid Analysis

Amino acid analysis was performed according to Mitchell et al. (Segata et al., 2012). Briefly, aliquots (20 μL) of plasma or QC were transferred to a micro-centrifuge tube containing 160 μL of 0.04 M sulfuric acid (with 15 μM L-norvaline as an internal standard). Samples were mixed and kept on ice for ~3 min. Sodium tungstate (20 μL of 10% w/v solution) was added to each sample and mixed immediately. Sample were incubated on ice (3 min) and centrifuged (14,000 rpm, 10 min). The supernatant containing the plasma amino acids was collected and transferred to a 1.5 mL micro-centrifuge tube.

For derivatization, 70 μL of 0.2 M borate buffer (1.24 g boric acid in 100 mL pH 8.8, adjusted with fresh 5M NaOH) was added to 10 μL of sample, standard or QC. 6-Aminoquinolyl-N-hydroxysuccinimidyl Carbamate (AQC) reagent (2.8 mg /mL dissolved in dry acetonitrile, 10 μL) was added to the sample, and the solution was vortexed mixed immediately. The derivatized sample was then transferred into the Ultra Performance Liquid Chromatography (UPLC) vial, capped and incubated at 55°C for 10 min. UPLC vials were then placed into a Ultra-high-performance liquid chromatography (UHPLC) system. The UPLC comprised a Thermo Fisher Scientific Dionex Ultimate 3000 pump, with an autosampler (maintained at 10°C), column oven (at 45°C), fluorescence detector (excitation wavelength: 250 nm, emission wavelength: 395 nm) (Thermo Fisher Scientific, Dornierstrasse, Germany). Separation was carried out on a Kinetex Evo 1.7 μm C18 100A 150 × 2.1 mm column that was preceded by a Krudkatcher inline filter (Phenomenex, Auckland, New Zealand). A mobile phase buffer (80 mM sodium acetate, 3 mM triethylamine, 2.67 μM disodium calcium ethylenediaminetetraacetic acid; pH 6.43) was run with a complex gradient of acetonitrile (ranging from 2% to 17% over 24 min). The flow rate of the UPLC was 0.55 mL/min. Chromeleon 7.1 software (Thermo Fisher Scientific) was used for data capture. The standard curves generated for each amino acid were used for calculating AA concentrations in the samples.

### Fecal Amino Acid Analysis

Fecal samples were collected from 41 individuals (20 very preterm and 21 terms). The samples were weighed (~100 mg) and lyophilized on a speed vac (Thermo Fisher Scientific Savant SC250EXP) overnight (24 h, 0.8 Hpa, with a −104°C refrigerated temperature trap). The wet and dry weight was noted for biomass normalization. Samples and quality controls (QC) were prepared by a tungstate precipitation. Briefly, 1,200 μL of 0.04 M Sulfuric acid (containing 15 μM L-Nor-Valine) was aliquoted into each tube containing dried sample. The mixture was vortexed (30 sec), sonicated (10 min), with repeating 2–3 times until there was a homogeneous mixture. Ceramic beads were added to samples that did not homogenize and vortexing and sonication treatment repeated. Sodium tungstate (150 μL of 10% w/v solution) was added to the homogenized samples on ice (3 min). Samples were centrifuged (20,800 g, 10 min, 4°C). The supernatant was then transferred into a 1.5 mL microfuge tube. The derivatization step was the same as for plasma samples (see above).

### Statistical Tests

Participants were recruited into two groups according to their gestational age: children born very preterm (i.e., ≤32 weeks of gestation) and children born at term (i.e., 37–41 weeks of gestation). Comparisons between gestational age groups were carried out using linear mixed models in SAS v.9.2 (SAS Institute, Cary, North Carolina). All models accounted for important confounding factors, mainly sex, ethnicity, birth weight SDS, gestational age, and maternal age. Age data are presented as means ± SD, whereas other data are means and 95% confidence intervals adjusted for the confounders in multivariate models.

A Core microbiome analysis (CORBATA) of 16S rRNA gene amplicon data was performed in R (version 3.5.0) using R and Perl scripts published in (Li et al., 2013,?). Non-parametric tests (i.e., Mann-Whitney Unpaired *t*-test between the very preterm and term groups) and Spearman correlations were performed on GraphPad Prism (version 7.03) using the operational taxonomic table (OTU) generated from the Ion Reporter software. Degree of difference in microbial relative abundances was determined using the Linear discriminant analysis (LDA) effect size (LEfSe) (Segata et al., 2011), a measure of statistical significance, with a cut off below −2 and above 2. Multivariate Association with Linear Models using MaAsLin (version 0.0.4) (Morgan et al., 2012) was performed to identify significant associations between microbial species and the participants' metadata.

## Data Availability Statement

Sequencing data is available from SRA project PRJNA628543. The datasets used and analyzed during the current study are available from the corresponding authors upon reasonable request.

## Ethics Statement

The studies involving human participants were reviewed and approved by Southern Health and Disability Ethics Committee (Ministry of Health, New Zealand 14/STH/90). Written informed consent to participate in this study was given by the parents of the children to provide samples.

## Author Contributions

TJ performed all sequencing and integrated analyses and wrote the manuscript. TV performed the phage analyses and wrote the manuscript. VC recruited subjects and performed clinical assessments. EA performed additional phage analyses. SJ and EM performed the SPME GC-MS. JD performed clinical statistical analyses. CE and WS contributed to the metagenomics. MB provided access to patients. ET performed the AA quantitation. DC-S, EF-B, PH, NR, GT, MV, and EA commented on the experimental design and commented on the manuscript. WC and JO'S designed the experiments, supervised VC, TJ, and SJ coordinated the study and wrote the manuscript.

## Conflict of Interest

The authors declare that the research was conducted in the absence of any commercial or financial relationships that could be construed as a potential conflict of interest.
