# Supplementary material for: Differences in Compositions of Gut Bacterial Populations and Bacteriophages in 5–11 Year-Olds Born Preterm Compared to Full Term
Source: Front Cell Infect Microbiol. 2020 Jun 16;10:276. doi: 10.3389/fcimb.2020.00276 (PMC7309444; doi:10.3389/fcimb.2020.00276)
Supplement: Supplementary file 1 [file Data_Sheet_1.PDF]

Supplementary results

Supplementary table 1. Demography and characteristics of the study population subgroup.

	Very Preterm Children	Term Children	*p*-value
**N**	20	21	
**Demography**			
Age (years) [1]	7.7 ± 1.5	8.6 ± 1.3	0.044
Sex ratio (males) [2]	65%	48%	0.26
Ethnicity (New Zealand European) [2]	75%	57%	0.23
**Birth characteristics**			
Birth weight SDS [1]	0.10 ± 1.04	0.25 ± 0.84	0.62
Gestational age (weeks) [1]	27.3 ± 2.2	40.2 ± 1.0	<0.0001
Delivery (C-section) [2]	45%	43%	0.89
**Infant characteristics**			
Breastfeeding [3]	95%	95%	0.99
**Anthropometry** [4]			
Weight SDS	0.00 (-0.41–0.41)	0.31 (-0.06–0.68)	0.25
Height SDS	0.26 (-0.19–0.72)	0.69 (0.26–1.12)	0.16
BMI SDS	0.02 (-0.44–0.48)	0.15 (-0.26–0.56)	0.66

Age data are means ± standard deviations; categorical data are n (%); [1]One-way ANOVA; [2]Chi-square test; [3]Fisher's exact test; [4]SDS outcomes: general linear regression models adjusted for sex, ethnicity and birth order, as well as mean parental BMI or mid-parental height. [†] Data on anthropometry is estimated marginal means and respective 95% confidence intervals, adjusted for confounding factors.

Supplementary table 2: Significantly different plasma volatiles in children born very preterm and at term.

Name	Mean ± SD (Very preterm)	Mean ± SD (Term)	q-value[¥]	Change[†]
4208633 2-Butanone, 1-2-furanyl-	53519 ± 12381	38080 ± 7967	0.000	Down
13466789 3-Carene	17057 ± 4418	10531 ± 3629	0.000	Down
71363 1-Butanol	92361 ± 38792	52583 ± 21297	0.000	Down
80568 alpha-Pinene	40730 ± 14042	27068 ± 6008	0.000	Down
110827 Cyclohexane	49040 ± 17271	30367 ± 13836	0.000	Down
108678 Mesitylene	71341 ± 40126	42689 ± 10780	0.001	Down
96377 Cyclopentane, methyl-	348760 ± 125461	229900 ± 88008	0.001	Down
100425 Styrene	152410 ± 86213	88446 ± 28656	0.001	Down
98828 Benzene, 1-methylethyl-	76756 ± 42669	47057 ± 12859	0.001	Down
1120214 Undecane	61875 ± 16310	48668 ± 9735	0.002	Down
124196 Nonanal	43506 ± 12382	34031 ± 6876	0.002	Down
96140 Pentane, 3-methyl-	148368 ± 51526	104531 ± 37015	0.003	Down
111717 Heptanal	78322 ± 46184	47815 ± 16913	0.003	Down
110430 2-Heptanone	62452 ± 24281	45451 ± 14422	0.003	Down
106423 p-Xylene (peak 2)	70931 ± 26078	50922 ± 21700	0.003	Down
616251 1-Penten-3-ol or Hexane, 2,2-dimethyl-	141929 ± 44169	103874 ± 40436	0.004	Down
95476 o-Xylene	269117 ± 105729	197044 ± 99878	0.005	Down
100414 Ethylbenzene or Benzene, 1,3-dimethyl- 99 percent	185795 ± 80661	125121 ± 62632	0.006	Down
106423 p-Xylene (peak 1)	232078 ± 95541	171901 ± 91243	0.010	Down
127913 beta-Pinene	31927 ± 5711	27459 ± 5193	0.010	Down
66251 Hexanal	1522569 ± 996001	947493 ± 608874	0.011	Down
28464417 2,2-Di2'-chloroethoxy-propane	8165 ± 2441	6185 ± 2478	0.011	Down
138863 Limonene	56174 ± 24421	40562 ± 9850	0.013	Down
638288 Hexane, 2-chloro- or 1-Hexene 90 percent	178827 ± 94651	118018 ± 56322	0.013	Down
142825 Heptane	80633 ± 33322	61045 ± 19369	0.013	Down
64197 Acetic acid	983438 ± 286463	791017 ± 210799	0.014	Down

3391864 1-Octen-3-ol	110972 ± 73021	75284 ± 40121	0.016	Down
3777693 Furan, 2-pentyl-	82104 ± 70081	50225 ± 29140	0.016	Down
108883 Toluene	209088 ± 69471	163510± 38596	0.025	Down
2216333 Octane, 3-methyl-	9141 ± 3136	13128 ± 6280	0.025	Up
107835 Pentane, 2-methyl-	214943 ± 56746	177265 ± 55722	0.032	Down
110543 n-Hexane	879350 ± 346628	680104 ± 235338	0.032	Down
107391 1-Pentene, 2,4,4-trimethyl-	111396 ± 35979	88714 ± 32912	0.034	Down
110930 5-Hepten-2-one, 6-methyl-	162575 ± 104073	117390 ± 60780	0.046	Down

¥FDR corrected p value

†Direction of change in term group

Supplementary table 3. Default module parameters used for 16s rRNA gene amplicon sequencing in Ion Reporter™ software (Version 5.12)

Parameter	Default value
**Primer(s) Detected**<br>Detect primers in each read at this location	Both ends
**Read Length Filter**<br>Filters out all reads shorter than the specified value (in base pairs) after trimming primers. For multiple amplicon experiments, use the size of the smallest amplicon (minus primer sequences). This filter is disabled when Primer(s) Detected is set to Both ends.	150
**Minimum Alignment Coverage**<br>Percentage value for the coverage needed between hit and query. Result hit alignment length from database needs to be at least this percentage of the query to be used. Calculated for each aligned read in the analysis.	90.0
**Read Abundance Filter**<br>Number of unique reads needed for that read to be a valid.	10
**Genus Cut-off**<br>Percentage identity value required to make a genus ID. Default value is 97.0%. Must be a smaller percentage value than used for species ID.	97.0
**Species Cut-off**<br>Percentage identity value required to make a species ID. Default value is 99.0%. Must be a larger percentage value than used for genus ID.	99.0

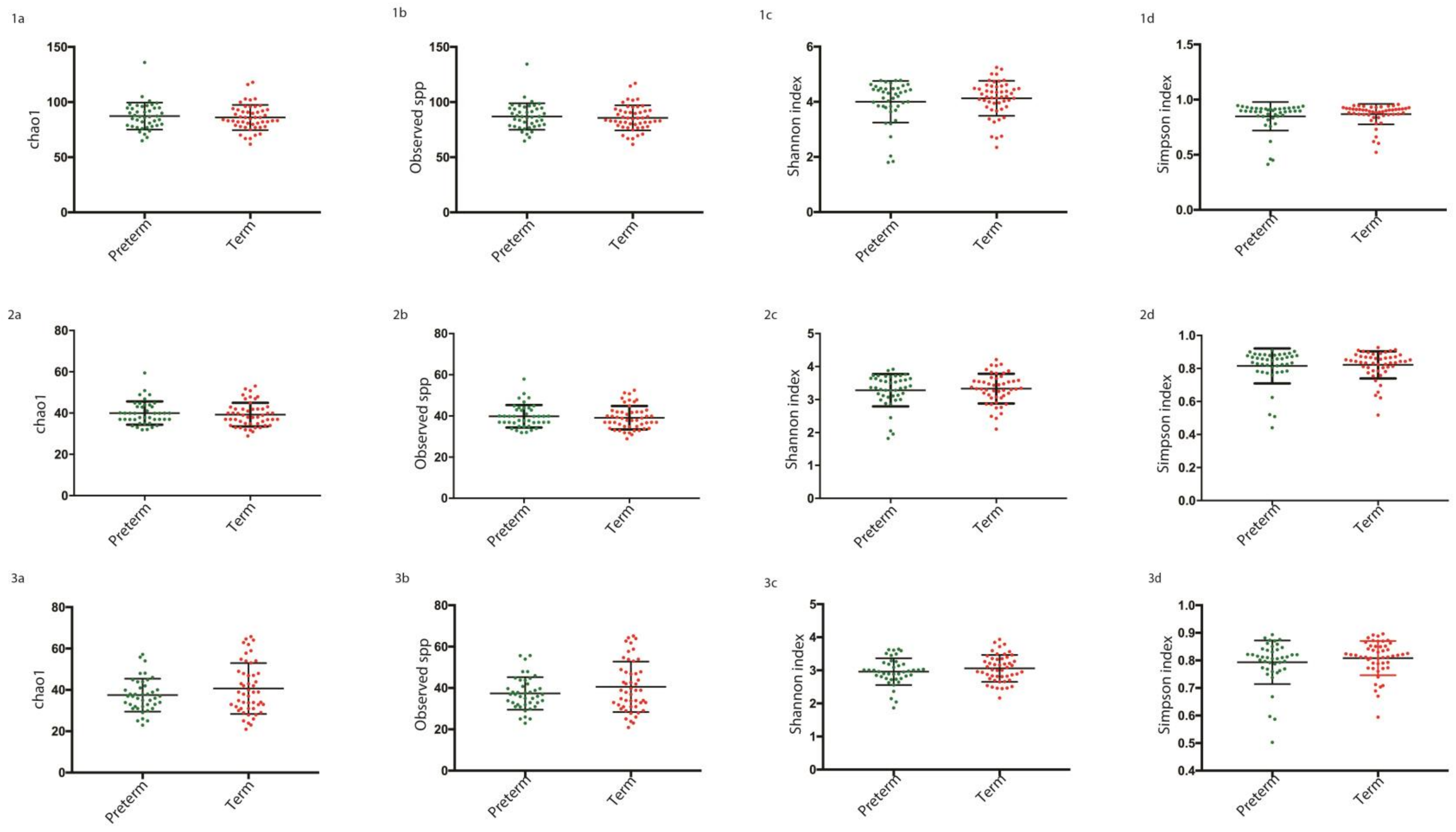

Supplementary fig. 1
Alpha diversity indices in children born very preterm (n=42) and at term (n=49). 1(a-d) alpha diversity indices at species level; 2(a-d) alpha diversity indices at genus level and 3(a-d) alpha diversity indices at family level.

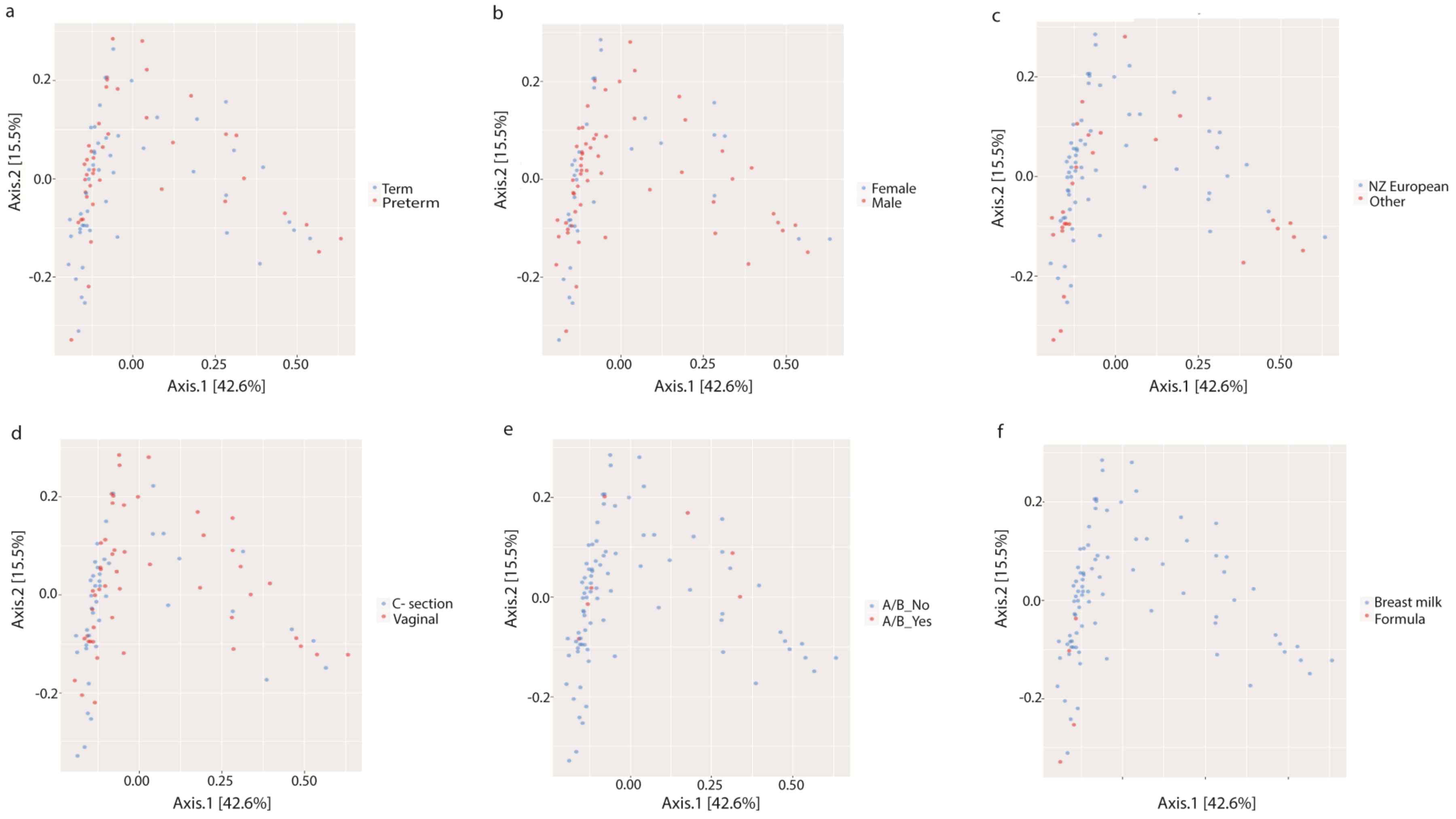

Supplementary fig. 2

Principle coordinates analysis (PCOA) based on the distance matrix of Bray-Curtis dissimilarity of the microbial community between (a) preterm vs term group; (b) female vs male; (c) NZ European vs other ethnic groups; (d) C-section born vs vaginal born; (e) Intake of antibiotic vs not and (f) breast milk and formula fed. This analysis is based on the microbial community obtained from the 16s rRNA gene sequencing data from the whole group (n=91).

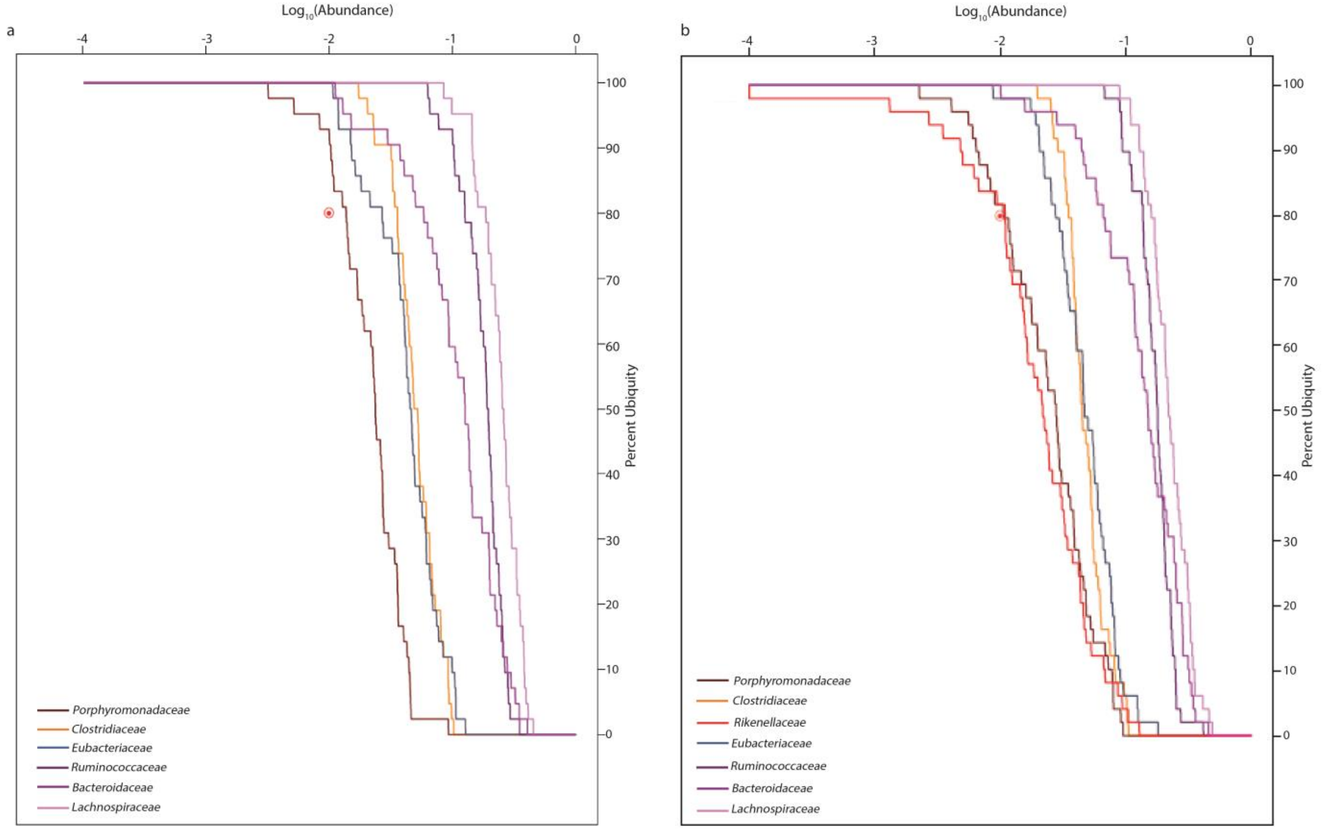

Supplementary fig. 3

The major core of gut microbiota of children born (a) very preterm (n=42) and (b) term (n=49). Above Ubiquity-Abundance plot was generated based on the cumulative distribution function (CDF) values for each bacterial family from 16S rRNA data with default cut-offs of ubiquity=0.8 and abundance 0.01. The y-axis represents the ubiquity percent (% of samples present) while the x-axis represents the abundance (log10 transformed). Each colored line represents a family that is above the specified cut-offs. The red "bull's eye" indicates where the cut-off is found. (a) Major core of microbiota of children very preterm consists of families (Ubiquity values at 0.01 abundance) *Clostridiaceae*=1.00, *Eubacteriaceae*=1.00, *Ruminococcaceae*=1.00, *Lachnospiraceae*=1.00, *Bacteroidaceae*=1.00, *Porphyromonadaceae*=0.93; (b) Major core of microbiota of children born at term include *Clostridiaceae*=1.00, *Eubacteriaceae*=0.98, *Ruminococcaceae*=1.00, *Lachnospiraceae*=1.00, *Bacteroidaceae*=1.00, *Porphyromonadaceae*=0.82 and *Rikenellaceae*=0.82.

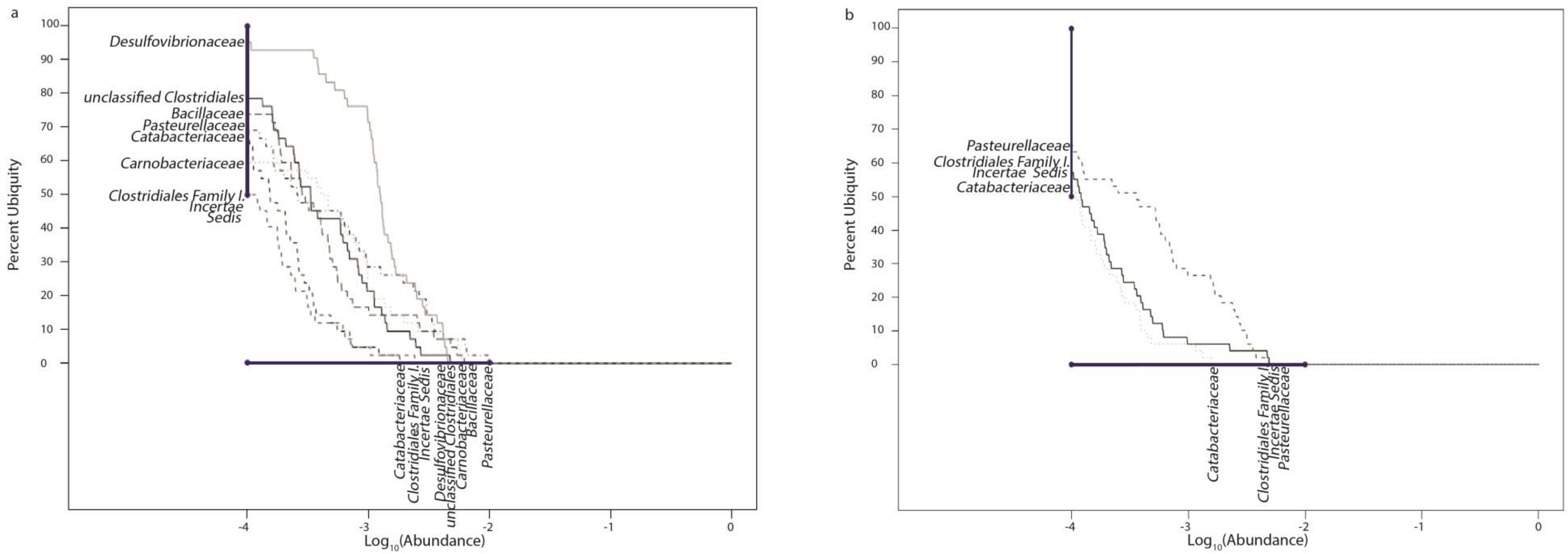

Supplementary fig. 4

The minor core of gut microbiota of children born (a) very preterm (n=42) and (b) term (n=49). The default cut-off values used are ubiquity=0.05 and abundance=0.01. Bacterial families which have ubiquity more than 0.5 and abundance less than 0.01 are considered as the part of the minor core. (a) Minor core microbiome of children born very preterm includes unclassified *Clostridiales Family I. Incertae Sedis*, *Carnobacteriaceae*, *Pasteurellaceae*, *Bacillaceae*, *Desulfovibrionaceae* and *Catabacteriaceae*. (b) Minor core microbiome of children born at term includes *Clostridiales Family I. Incertae Sedis*, *Pasteurellaceae* and *Catabacteriaceae*.

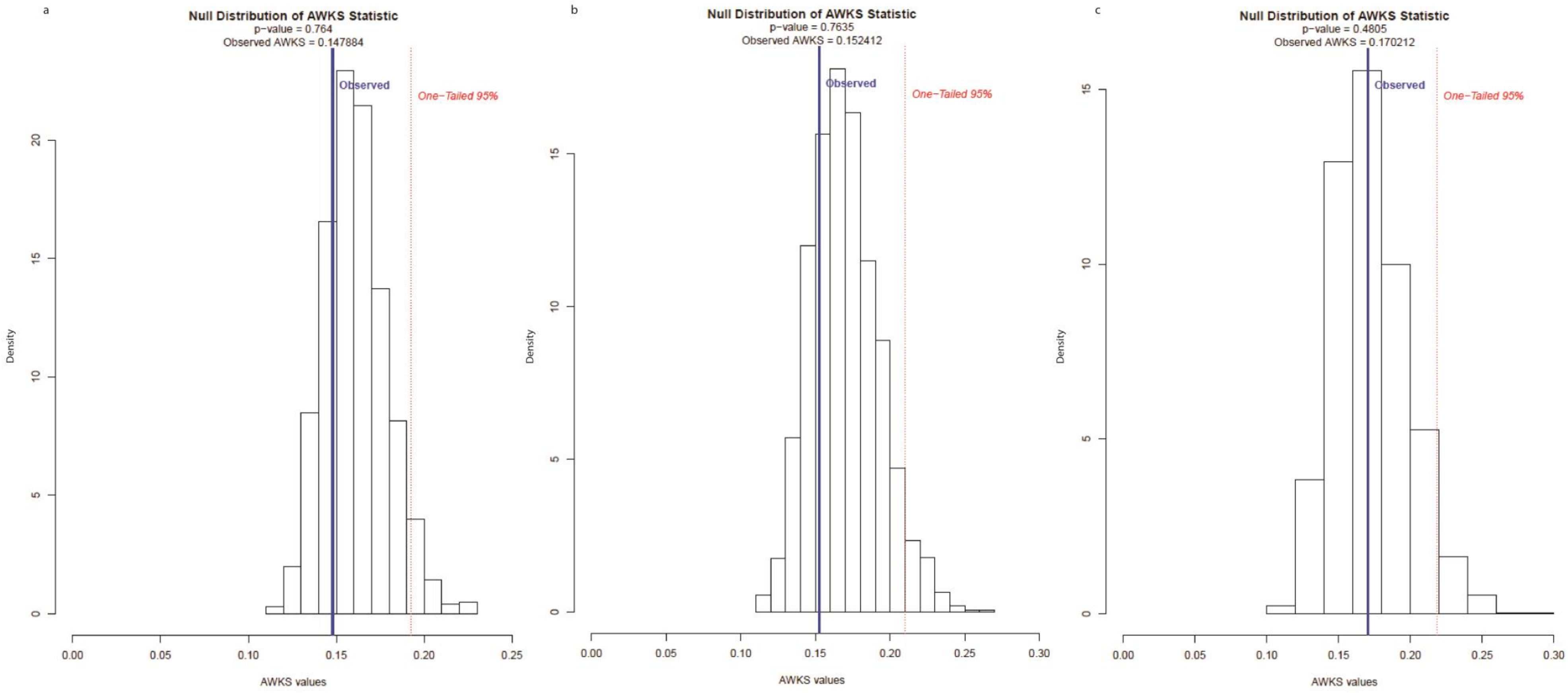

Supplementary fig. 5

The magnitude of the difference between the taxonomic profiles of two groups [very preterm (n=42) and term (n=49)] using the Abundance-Weighted Kolmogorov-Smirnov (AWKS) statistic at (a) species, (b) genus and (c) family levels. Larger AWKS values represent the greater differences between the two cohorts. Two cohorts were not significantly different at a *p*-value of 0.05.

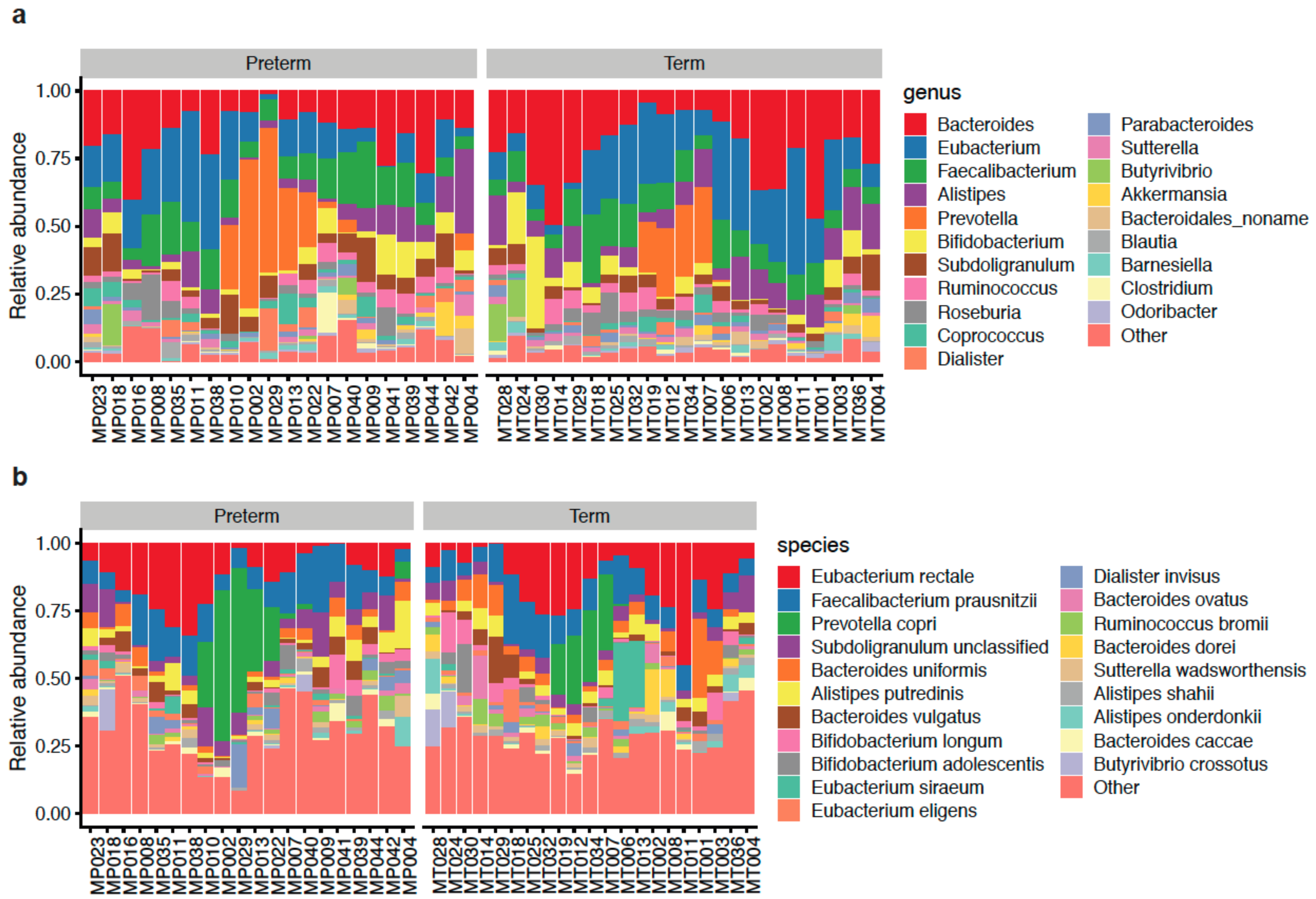

Supplementary fig. 6.

Relative abundances of top 20 most abundant bacterial (a) genera and (b) species according to the shotgun metagenomic data.

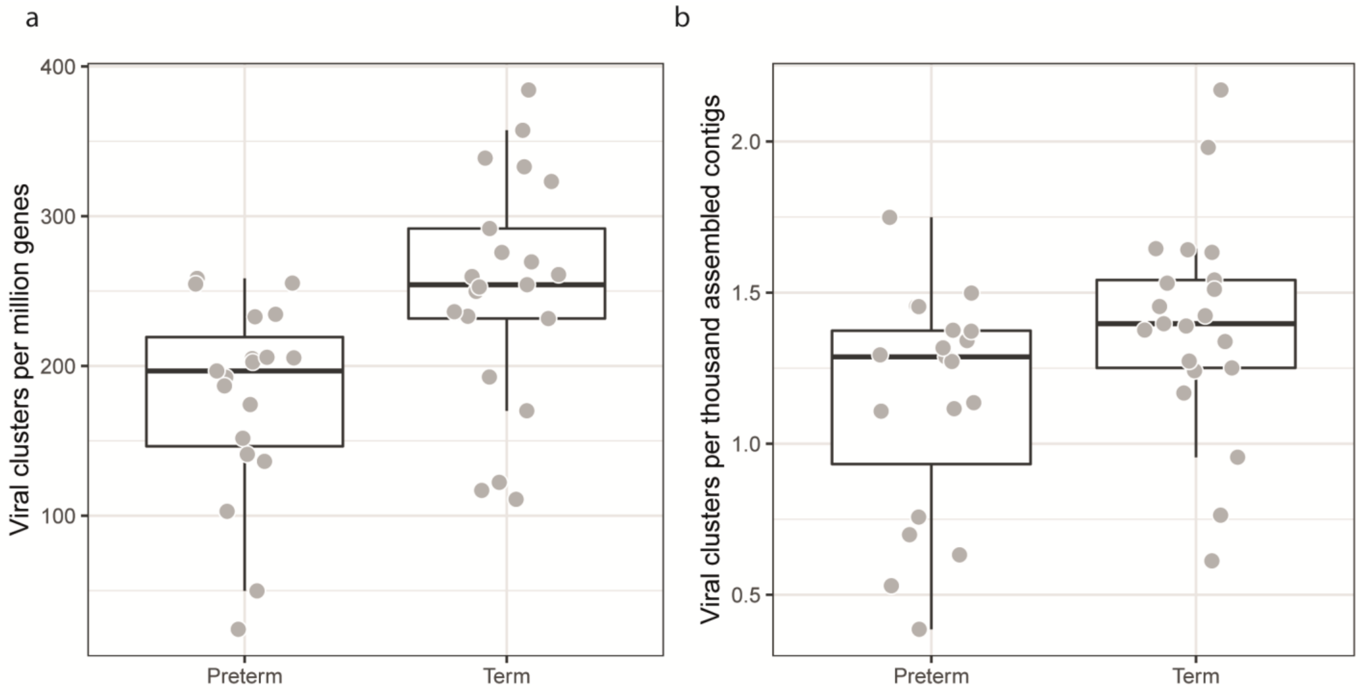

Supplementary fig. 7.
Differences in viral cluster richness mirrored that seen in phage richness. (a) Viral clusters measured per million genes in children born very preterm and term, respectively. (b) Viral cluster richness measured per thousand assembled contigs.

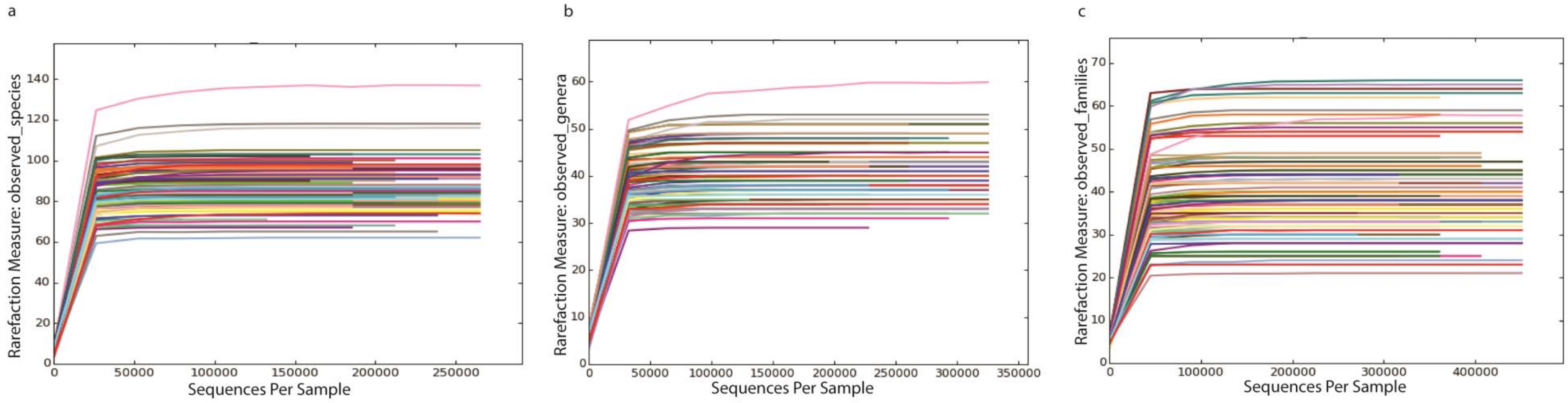

Supplementary fig. 8.

Rarefaction curves for observed species at (a) species, (b) genus and (c) family level. Curves were calculated for all samples (n=91).
