# Supplementary material for: Differences in Compositions of Gut Bacterial Populations and Bacteriophages in 5–11 Year-Olds Born Preterm Compared to Full Term
Source: Front Cell Infect Microbiol. 2020 Jun 16;10:276. doi: 10.3389/fcimb.2020.00276 (PMC7309444; doi:10.3389/fcimb.2020.00276)
Supplement: Supplementary file 2 [file Table_1.DOCX]

**Table 1.** Demography and summary characteristics of the study population.

|  | **Very preterm**  **Children** | **Term**  **Children** | ***p*-value** |
| --- | --- | --- | --- |
| **N** | 51 | 50 |  |
| **Demography** |  |  |  |
| Age (years) ^1^ | 7.8 ± 1.3 | 8.3 ± 1.4 | 0.034 |
| Sex ratio (females) ^2^ | 20 (39%) | 19 (38%) | 0.90 |
| Ethnicity (New Zealand European) ^2^ | 39 (76%) | 30 (60%) | 0.074 |
|  |  |  |  |
| **Birth characteristics** |  |  |  |
| Birth weight standard deviation score (SDS) ^1^ | 0.42 ± 0.91 | 0.32 ± 0.90 | 0.60 |
| Gestational age (weeks) ^1^ | 28.1 ± 2.2 | 39.9 ± 1.2 | <0.0001 |
| Delivery (C-section) ^2^ | 30 (59%) | 18 (36%) | 0.021 |
|  |  |  |  |
| **Infant characteristics** |  |  |  |
| Breastfeeding^3^ | 47 (92%) | 48 (96%) | 0.68 |
|  |  |  |  |
| **Childhood anthropometry** ^4†^ |  |  |  |
| Weight SDS | -0.16 (-0.49–0.10) | 0.47 (0.21–0.73) | 0.0005 |
| Height SDS | 0.31 (0.03–0.58) | 0.92 (0.67–1.17) | 0.0006 |
| BMI SDS | -0.20 (-0.40–-0.01) | 0.29 (0.09–0.49) | <0.0001 |
|  |  |  |  |

Age data are means ± standard deviation; categorical data are n (%); ^1^One-way ANOVA; ^2^Chi-square test; ^3^Fisher's exact test; ^4^SDS outcomes: general linear regression models adjusted for sex, ethnicity and birth order, as well as mean parental BMI or mid-parental height. ^†^ Data on anthropometry is estimated marginal means and respective 95% confidence intervals, adjusted for confounding factors.
